# The impact of COVID-19 on Physical Activity of Czech children

**DOI:** 10.1371/journal.pone.0254244

**Published:** 2021-07-08

**Authors:** Tereza Štveráková, Jakub Jačisko, Andrew Busch, Marcela Šafářová, Pavel Kolář, Alena Kobesová

**Affiliations:** 1 Department of Rehabilitation and Sports Medicine, Postgraduate Medical School, Second Faculty of Medicine Charles University and Motol University Hospital, Prague, Czech Republic; 2 Health and Human Kinetics, Ohio Wesleyan University, Delaware, OH, United States of America; Nanyang Technological University, SINGAPORE

## Abstract

**Introduction:**

The pandemic of coronavirus disease (COVID-19) and related restrictions (closed schools and sports centers, social isolation, masks) may have a negative impact on children’s health. The purpose of this study was to evaluate the level of physical activity (PA) of Czech children during COVID-19 in autumn 2020.

**Methods:**

Ninety-eight Czech children (mean age = 10.1 ± 1.47 years) completed the standardized Physical Activity Questionnaire for Older Czech Children (PAQ-C/cz) during COVID lockdown. Data were compared with previously published norms. Thirty-five children also reported daily number of steps measured by accelerometers.

**Results:**

Total PAQ-C score was 0.38 lower during COVID compared to Pre-COVID [*t*(302) = 5.118., *p* < .001]. The male PAQ-C total score was 0.37 lower [t(146) = 3.21., p = .002)] and the female total score was 0.39 lower [t(154) = 3.97., p < .001] during COVID compared to Pre-COVID. Specifically, responses of PA during spare time, before-school, physical education (PE), and recess were significantly lower during COVID. The average number of steps was 7.767 steps/day (boys = 9.255; girls = 6.982).

**Conclusion:**

COVID lockdown resulted in significant reduction of PA in Czech children. Strategies to promote adequate PA of children during the pandemic need to be determined.

## Introduction

Healthy physical development in children is largely dependent on sufficient physical activity (PA), reduced sedentary behavior (SB) and adequate sleep. These three factors are referred to as a movement behavior [1]. According to the World Health Organization (WHO), lack of regular PA and increased time spent in sedentary activity are globally the fourth highest risk factor attributed to mortality, with overweight and obesity being the third leading risk factor of mortality in middle and high-income countries, behind only high blood pressure and tobacco use [2, 3].

Regular PA promotes general health, prevents obesity and other civilization diseases [3]. To meet the criteria of the optimal movement behavior, it is recommended that children and adolescents aged 5-13 years strive to achieve a daily minimum of 60 minutes of moderate to vigorous PA, limit sedentary recreational screen time to 2 hours maximum, and acquire 9 to 11 hours of uninterrupted sleep per night [1, 4].

Beginning around six years of age, children undergo significant psychosocial developmental changes reflecting their self-concept and relationship with the environment [5]. An important part of their daily routine and healthy lifestyle is their regular school attendance and organized PA [5–8]. It contributes to the improvement of social contacts and can influence quality of life. There is also strong evidence promoting the effectiveness of regular PA and exercise in the treatment of depression, anxiety and improving mental well-being [9].

Many countries have successfully worked towards these goals of optimal children’s movement behavior by implementing different organized sport activities and integrating regular physical education (PE) lessons in schools. However, in December 2019 the first cases of the coronavirus disease 2019 (COVID-19) were reported and on January 30 COVID-19 became officially declared international public health emergency [10]. Governments implemented restrictions involving school and sport grounds closures resulted in health risks behaviors especially reduced PA and increase SB [11]. The regulations had negative effect on various mental and health aspects in children and youth such as increasing obesity [12, 13], pain [14], depression, anxiety, loneliness feelings [15–17], sleep disturbances [18, 19], decreased cardiorespiratory fitness [20] and many others, affecting especially socio-economic deprived children [11].

PA in Czech children and youth was reported to be insufficient already before the COVID. Only 35% of children population performed the recommended amount of PA, i.e. 60 minutes of moderate to vigorous PA per day. The high rates of excessive screen-time were reported by Gaba et al. in study collecting data during the pre-pandemic 2018 year. Organized PA and sport have been performed by 55% of girls and 70% of boys, joint sports activities with the family at least once a week were reported only by 34% of girls and 37% of boys. Under normal conditions all schools in the Czech Republic must guarantee at least 90 min per week of PE [21]. However, this amount of time is considered insufficient and there has been a long-standing political and professional discussion on increasing PE classes at primary schools. Most schools offer more PE classes than the mandatory 90 minutes per week and provide favorable environment to promote PA outside PE classes. Still, there has been a call for increase of PE hours in elementary schools and after-school health-enhancing PA promotion [21, 22].

The first three cases of COVID-19 were reported in the Czech Republic on March 1^st^, 2020 [23]. Outbreak of COVID-19 resulted in full shutdown of organized sports and public sports facilities in the Czech Republic. From March 2020 till May 2021 school attendance and organized sport activities have been largely unavailable in the Czech Republic. By April 2021, the Czech Republic has recorded the second highest confirmed death rate in the world with 1.8% case fatality and 282.14 deaths per 100 000 people [24]. With just a shortbreaks, children were ordered to stay at home, online education was established, and organized sport activities were prohibited both indoor and outdoor. Such long lasting restrictions may create unintended poor habits of decreased PA and increased SB in child populations [25], having negative effects on daily routines and opportunities for being active [26].

Movement activities of children and adolescents during COVID differ among countries due to different policy restrictions and the number of COVID-19 infections [26]. According to UNESCO, from all European Union (EU) countries, the Czech Republic had the longest school closure during the pandemic, that is 42 weeks [27]. Even countries with much shorter period of school closure, such as France (11 weks school closure) [28] or Portugal (24 weeks closure) [29] or Spain (15 weeks closure) [30] confirmed decreased levels of PA in children during COVID calling for the development of effective national action. German study where the schools were closed for 30 weeks reports decreased sports activity but increase in habitual physical activities such as gardening, housework, cycling or walking [26]. Other studies also report the shift of PA towards nonorganized outdoors activites such as walking, running, bycycling and alike [19, 31]. This study attempts to analzye the impact COVID-19 has on PA on children in the Czech Republic, where COVID death rates were very high and the schools and sport facilites closed for the longest period of time in the whole EU.

It is difficult to determine the most appropriate tools to evaluate physical behavior aspects in children and the "gold standard" is still not available [4, 32–34]. Despite a certain degree of bias, questionnaires and accelerometers are currently the most widely used methods to collect such data. The principle of self-assessment questionnaires is based on the respondent’s ability to recall his or her activities during the observed period of time, usually one week or one month back. Although only some questionnaires demonstrate adequate validity and reliability, they represent a cheap and easy way to assess the amount of PAs [1, 35, 36].

The Physical Activity Questionnaire (PAQ) is one of the most frequently used questionnaires worldwide [37]. The Physical Activity Questionnaire for Older Children (PAQ-C) variant was developed for children aged 8–14 years old [32] and was recently standardized for Czech children by Cuberek et al. [38]. PAQ-C is considered a reliable tool to evaluate children’s PA [5, 38–40]. Questionnaires can be combined with data obtained from accelerometers that monitor the amount of PA. Although accelerometers do not capture certain types of PA accurately (such as cycling), they can provide a rough estimate of the level of PA achieved by the subject throughout the day [35].

This is the first study comparing PA of school children during the COVID lockdown time with pre-COVID norms defined by the Czech version of PAQ-C (PAQ-C/cz) and by evaluating their number of daily steps.

## Methods

### Study design

An anonymous survey to evaluate PA of children aged 8–12 years was conducted during COVID Lockdown in the Czech Republic in November and December 2020. The data were compared with the Pre-COVID norms defined by the same questionnaire (PAQ-C/cz) (see the S1 and S2 Files). The subjects were also asked to monitor and report the number of daily steps if they have appropriate measurement device (smart watch or smart phones) available. The study was approved by the Ethics Committee of the University Hospital Motol and 2^nd^ Faculty of Medicine, Charles University in Prague (EK-1730/20).

### Participants

Our data were collected during COVID lock down from November 2020 to January 2021. It was compared with the norms collected by Cuberek et al. [38] one year earlier, during the same time of the year before the COVID-19 occurred in the Czech Republic. Participants were recruited using an information leaflet created for the purpose of this study. It was published either electronically on an official Dynamic Neuromuscular Stabilization (DNS) website www.rehabps.com or in paper form at physiotherapy centers and at the Motol University Hospital. It contained information on the purpose of the study, a standardized questionnaire and the informed consent. Participation in the study was voluntary and the informed consent was signed by the participant’s parent or legal representative. In total, ninety-eight children participants (56 girls and 42 boys) completed the questionnaire either electronically or as a hard copy. Inclusion criteria for participation comprised age (8–12 years) and participation in distance learning education. Participants were excluded if they had any serious health condition. The questionnaire data revealed participants in this cohort (98), were living in cities and villages of different sizes, came from different elementary schools, and came from various family and social backgrounds. Table 1 shows anthropometric characteristics of the study participants.

**Table 1:** Study characteristics comparing Cuberek et al. [38] Pre-COVID data with during COVID data of Czech children.

	Sex	Sample Size	Age*	BMI*
Cuberek et al Pre-COVID cohort	Male	106	11.08 (0.84)	18.46 (3.13)
	Female	100	11.17 (0.82)	17.36 (2.68)
	Total	206	11.13 (0.83)	17.92 (2.97)
During COVID cohort	Male	42	10.21 (1.49)	17.56 (3.06)
	Female	56	10.02 (1.46)	17.20 (2.70)
	Total	98	10.10 (1.47)	17.35 (2.85)

*Reported as mean (standard deviation), note: BMI: Body mass index.

### Physical Activity Questionnaire for Older Children (PAQ-C/cz)

A validated Czech version of the standardized PAQ-C/cz was used to evaluate the level of PA in the observed cohort. The standardized PAQ-C was recently adapted into the Czech version by Cuberek et al., which assessed its psychometric properties and recommended it as a tool for physical activity assessment in large-sample research studies [38].

PAQ-C/cz is a ten-item, self-administered, seven-day recall questionnaire for children 8–14 years old. The questionnaire provides a summary of PA calculated from nine items, each scored on a five-point scale (with 1 representing the lowest level and 5 representing the highest level of PA). The total PAQ-C score is calculated as a mean value from the nine different item scores including: children´s spare time activity (question 1), activity before school (question 2), activity during physical education (PE) lessons (question 3), activity during recesses (question 4), activity after school (question 5), activity in the evening (question 6), activity during weekend (question 7), statement of free time activity during last week (question 8), and activity level and frequency performed each day during last week (question 9). Question number 10 is of a qualitative character inquiring about any disease or other obstacles to perform PA during the observed period of time and therefore cannot be included in the final score calculation [38].

Because the survey was done at a time of distance online school education, there was a note in the questionnaire form to evaluate PA during recesses as the time between online lessons. Collected COVID data were compared with the Pre-COVID norm data recently published by Cuberek et al. [38]. See Table 1 to compare demographic characteristics of COVID and Pre-COVID cohorts. Additionally, thirty-five children from our COVID cohort reported daily number of steps using smart watch or smart phones to count the steps. Children provided a print screen from the device to prove the number of steps for each day.

### Statistical analysis

Descriptive statistics were calculated for all variables. Data are mean ± standard deviation, unless otherwise stated. Independent-samples t-tests (2-tailed) were performed to assess differences in PA from the PAQ-C scores among Czech children between the period of COVID lockdown with published PA norms prior to COVID restrictions. Statistical significance was determined a priori at *P* < 0.05 for the PAQ-C total score. When comparing responses to individual questions within the PAQ-C, Bonferroni corrections were utilized to reduce chances of Type 1 error, and was set at *P* < 0.005. Power analysis, using G*Power 3.1, indicated 128 subjects were needed (64 per group) to detect a medium effect size of 0.5 and an achieved power of 0.80. Effect sizes were interpreted as very small (< 0.2), small (0.2–0.5), medium (0.5–0.8), or large (> 0.8) [41]. Data analyses were conducted using the Statistical Package for the Social Sciences v27 (SPSS Inc, Chicago, IL).

## Results

Distribution of the 98 participants during COVID lockdown were: males (n = 42, 42.9%), females (n = 56, 57.1%) and the 206 participants included from pre-COVID data were: males (n = 106, 51.5%), females (n = 100, 48.5%). Participant characteristics for both COVID lockdown and pre-COVID data are outlined in Table 1. Not all data was normally distributed, as assessed by Shapiro-Wilk’s test. Due to the robustness of the independent samples t-test, data was not altered. Cronbach‘s alpha scores were calculated to score internal consistency for both sets of PAQ-C/cz questionnaire data (pre-COVID and COVID lockdown) using all nine questions. Cronbach’s alpha for pre-COVID questionnaire data (Cuberek et al. [38]) was acceptable at 0.758, and COVID lockdown Cronbach’s alpha was interpreted as good at 0.806 [42]. Results of all independent samples *t*-tests with 95% confidence intervals are presented in Table 2, with gender-specific data presented in Table 3. Significant differences were found in the mean PAQ-C total scores between pre-COVID and COVID lockdown, *t*(302) = 5.118., *p* < .001, *d =* .*63*, with a mean difference of .385 (95% CI: .237, .532).

**Table 2:** Comparison of Czech children scores on the PAQ-C regarding PA before and during COVID pandemic (mean [standard deviation]).

Measure	Cuberek et al. Pre-COVID (n = 206)	COVID Lockdown (n = 98)	Mean Difference (95% CI)	Effect Size	*P* V*alue*
Total PAQ-C Score	2.69 (0.59)	2.30 (0.66)	0.38 (0.24, 0.53)	0.63	< .001*
Q1 Spare time activity	1.34 (0.22)	1.26 (0.17)	0.07 (0.03, 0.12)	0.38	.001**
Q2 Before-school activity	2.06 (1.37)	1.63 (1.08)	0.43 (0.15, 0.72)	0.34	.003**
Q3 Physical education	3.83 (1.15)	2.26 (1.37)	1.57 (1.26, 1.89)	1.28	< .001**
Q4 Recesses	2.82 (0.95)	1.87 (1.03)	0.95 (0.71, 1.18)	0.97	< .001**
Q5 After-school activity	3.00 (1.11)	3.14 (1.19)	-0.14 (-0.42, 0.14)	-0.12	0.32
Q6 Evenings	2.59 (1.07)	2.43 (1.30)	0.16 (-0.11, 0.44)	0.14	0.25
Q7 Weekend	2.90 (0.98)	2.82 (0.92)	0.08 (-0.15, 0.31)	0.09	0.49
Q8 Statement	2.71 (1.04)	2.65 (1.10)	0.06 (-0.19, 0.32)	0.06	0.64
Q9 Weekly activity	2.93 (0.76)	2.70 (0.85)	0.23 (0.04, 0.43)	0.30	0.016

*Statistically significant difference observed (*P* < 0.05).

**Statistically significant difference observed (Bonferroni correction *P <* 0.005).

Note: PAQ-C: Physical Activity Questionnaire for Older Children.

Values are tabulated scores from PAQ-C.

Effect size = calculated Cohen’s d.

**Table 3:** Gender specific scores on the PAQ-C before and during COVID pandemic mean [standard deviation]).

Measure		Cuberek et al. Pre-COVID	COVID Lockdown	Mean Difference (95% CI)	Effect Size	*P* V*alue*
Total PAQ Score	Male	2.69 (0.62)	2.32 (0.69)	0.37 (0.14, 0.61)	0.59	.002*
	Female	2.68 (0.56)	2.29 (0.64)	0.39 (0.20, 0.58)	0.66	< .001*

*Statistically significant difference observed (Bonferroni correction *P <* 0.025).

Note: PAQ-C: Physical Activity Questionnaire for Older Children.

Values are tabulated scores from PAQ-C.

Effect size = calculated Cohen’s d.

After a Bonferroni correction, independent samples *t*-tests compared answers on nine individual questions of the PAQ-C. Significant differences between pre-COVID and COVID lockdown mean scores were noted for: Spare time (Q1) *t*(239.2) = 3.39., *p* = .001, *d* = .38, before school (Q2) *t*(236.9) = 2.97., *p* = .003, *d* = .34, PE (Q3) *t*(164.87) = 9.85., *p* < .001, *d-* = 1.28, and recesses (Q4) *t*(302) = 7.91., *p* < .001, *d* = .97. No significant differences were noted for: After school (Q5) *p* = 0.32, evenings (Q6) *p* = 0.25, weekend (Q7) *p* = 0.49, statement (Q8) *p* = 0.64, or weekly activity (Q9) *p* = 0.16. See S1 Graph. There were no differences noted between genders when comparing PAQ-C total scores pre-COVID or during COVID lockdown. After dichotomizing COVID lockdown data into younger (8–9 yr., n = 43) and older (10–12 yr., n = 55) groups, no differences were noted between PAQ-C total scores (*p* = .217).

## Discussion

The COVID-19 pandemic has created unprecedented situations by which strict community lockdowns have negatively affected the movement and health behavior in children [11]. Negative consequences like increasing obesity [12, 13], pain [14], depression, anxiety, feelings of loneliness [15–17], and sleep disturbances [18, 19] are closely related to PA levels [31, 43, 44]. Our findings are similar to others [28, 30, 45] demonstrating a significant decline in PA in children during COVID compared to pre-COVID. This problem is quite complex however, due to the multifactorial nature of various changes in PA during COVID such as: social status and family income [19], city dwelling versus villages or suburbs [44], parental education levels [18], pre-pandemic sports habits [46], the level of national restrictions, and age [26] among others.

Studies suggest of a relationship between age and the amount of PA during lock down [26, 30]. Speculating that older children could be more prone to online games, watching movies and following the social networks than younger children, we compared the COVID data for younger (8–9 yrs, n = 43) and older (10–12 yrs, n = 55) groups, but no differences were noted between PAQ-C total scores. Today, unorganized free-play activities have become less common and children’s time has been increasingly devoted to organized PA [47]. Due to COVID-19 regulations, even the youngest school children significantly reduced their PA and did not replace the regular amount of movement with any alternative PA such as playing in the garden, in the park, or running around the house. This was true both for the boys and the girls. We have not confirmed the increase in habitual outdoor PAs like other studies [26, 44]. A recently published study by Ng et al. on Czech adolescents’ remote school and health experiences during spring lockdown reports more PA. This discrepancy is perhaps due to age differences since our study evaluates PA in children 8–12 years old, while the study by Ng was done on older children aged 11–15 years at a different time of year. We collected data in November-January, while Ng collected their data from May-June, when more individual outdoor PA could be expected [48].

Organized sports of all kinds, both indoor and outdoor were significantly reduced in our respondents (Q1-spare time activity) as well as morning, i.e. before school activities (Q2). This is not surprising, since children did not walk to school and could not participate in regular sports because playgrounds and sports clubs were closed. The results of our survey also show that PE activities (Q3) were significantly limited as well. This should be considered and possibly changed by the PE teachers. If other school subjects can be taught online, there is no reason why PE could not. We can assume that almost every child can do simple exercises such as jumps, push-ups, sit-ups, plyometric exercises at home under the online guidance of the teacher. At the same time the PE teacher can motivate children and request to do individual activities such as running, walking, nordic-walking, scootering, bicycling and alike recording the frequency and intensity in a PA diary that should be signed by the parents and regularly presented to PE teacher. The same is true for the PA during recesses which were also reported significantly low. PE teachers could possibly take over during recesses to guide children through simple stretching exercises and repetitive aerobic movements under their online guidance to compensate for the SB. Strategies and recommendations for PE via distance learning have already been discussed in the literature confirming PE teachers’ critical role in supporting student health during the COVID-19 pandemic [49] but the conditions differ significantly by country and local policies [50]. Unfortunately, this type of regime was not established in many Czech schools. Children in the questionnaire mostly responded “I did not have a PE lesson/I did not do PE”. Also, the Czech pre-COVID PE score was rather low compared to other countries including data from Turkey, Great Britain, and China. In Turkey it was 4.52 ± 0.99 [51], two surveys in Great Britain reported a PE score of 4.14 ± 0.80 and 4.18 ± 0.74 [52] and in China 4.04 ± 0.98 [53]. The Czech pre-COVID PE score was 3.83± 1.15 and during COVID it was only 2.26 ± 1.37 [38].

There were no significant differences in after-school activities, evening, or weekend activities. We speculate this results from parental care motivating children and establishing routines for movement at a time when the family is together [30]. The most frequently reported PA during the period of quarantine restrictions was tourism and walking, followed by bicycling and athletics, specifically running (see S2 Graph). The weekly activities mean score was lower during-COVID compared with pre-COVID, but not enough to be statistically significant. The most active weekday was Saturday for both males and females, which is in line with other studies underlying the importance of accessibility to outdoor spaces for sufficient PA during the pandemic [19, 26, 44].

Tourism has a strong tradition in the Czech Republic, and likely represents the main type of regular PA during pandemic both for children and adults. COVID-19 associated regulations may change the structure of general population PA preferring the outdoor PA. Tourism is an optimal form of PA for the whole family and parental support is an important correlate of children’s PA [19, 54]. The data from PAQ-C are in line with data obtained from the thirty-five subjects from our cohort who also reported the number of daily steps measured by the pedometers (smartphones, watch). The average number of daily steps was 7.767 steps with boys reporting 9.255 steps per day and girls 6.982 per day on average. The highest number of steps (10.244 steps on average) was measured on Saturday. Still, Czech children during COVID-19 do not meet the recommendations for the number of daily steps. Suggested number of steps for normal 6–12 years children population to maintain good health ranges from 12.000 to 16.000 steps/day [55–57]. For effective reduction of childhood obesity, the girls are recommended to take at least 11.000 and the boys at least 13.000 steps/day five days per week at minimum [58]. According to Vuković et al., children who were physically active before the pandemic tend to continue their activities during the emergency state [46]. The insufficient amount of PA of Czech children before COVID became even more pronounced during COVID [21].

When comparing pre-COVID PAQ-C scores of Czech children with children of different countries, several differences exist. A Turkish study applied the PAQ-C survey to 784 primary school students (ages 9-14 years) and reported total PAQ-C scores to be 3.16 ± 0.73 [51]. A study in the United States performed the PAQ-C survey in a group of 1,172 children and noted differences when separated by race: European-American (3.36 ± 0.80), African-American (3.37 ± 0.69), and Hispanic (3.19 ± 0.64) [59]. Two British studies reported mean PAQ-C scores of 3.49 ± 0.68 (n = 336) and 3.36 ± 0.67 (n = 131) [52]. For Chinese children (n = 742), the total PAQ-C score was lower, 2.62 ± 0.68 [53]. The reported total PAQ-C score for the pre-COVID Czech population was only 2.69 ± 0.59, which means Czech children move less than Turkish, US and British children. Only Chinese children move slightly less then Czech. This is an alarming finding that even under normal conditions Czech children do not move sufficiently. The current findings of this study demonstrate a decrease in an already rather sedentary population of Czech children, which can only worsen as COVID lockdowns prolong.

Sufficient PA is critical in civilization disease prevention [60–62]. Children should be physically active daily as part of play, games, sports, transportation, recreation, PE, or planned exercise in the context of family and if possible in the context of school and community [62]. It seems that most families tried to compensate the lack of PA during COVID lockdown by tourism, especially on weekends. However, walking can be effective compensation only if optimal duration, speed, frequency, cardiorespiratory level, postural-stabilization and other parameters are respected [61, 62]. Especially the gait duration, speed and country terrain (hilly versus flat) is critical for sufficient oxygen uptake and aerobic fitness. For health benefits school-aged children and youth should accumulate at least 60 min of moderate to vigorous PA on a daily basis [61–63]. More daily PA provides greater health benefits [62]. To meet such criteria, brisk walking, jogging or hiking in nature is a good variant [61, 64]. We do not know the parameters of the reported tourism, and therefore cannot tell if it was an effective compensation for PA.

When analyzing both during-COVID and pre-COVID PA, the aspect of weather should be taken into account. The comparison of our data collected during COVID lockdown was coincidentally collected during the same months (November—January) as the pre-COVID data reported by Cuberek et al. just one year prior [38]. Rain, temperature, and earlier times of dusk may discourage children from doing outdoor activities. The autumn season is characterized by a decrease in energy expenditure in children attaining lower numbers of steps per day [65]. Perhaps spring and summer lockdowns would have less significant effects on children’s PA.

Studies mapping the level of PA during COVID time in other countries exist, but other methods than PAQ-C were applied. An American study monitored the time spent by eleven common types of PA (walking, running, swimming, etc.) and twelve common types of SB (watching television, playing computer games, reading, etc.). The most common types of PA during the early-COVID-19 period was unorganized play and unstructured activities such as running around, hide and seek and similar games (90% of children) or going for a walk (55% of children). Parents of older children (9-13 years) admitted greater decreases in PA and greater increases in SB than parents of younger children (5–8 years) [66]. This was not confirmed by our study, because no differences were noted between younger and older children PAQ-C total scores (*p* = .217). The Canadian online study with children aged 12–17 evaluated PA, SB and sleep time during the March 2020 COVID-19 pandemic. Canadian children and youth had lower PA levels, less outdoor time, higher SB (including leisure screen time), and more sleep during the outbreak [67]. A Portuguese anonymous online survey examined children aged up to 12 years at the end of March 2020. During COVID boys and girls performed PA equally but children with a previous routine of outdoor activities and children with siblings were more active. However, the total time spent being PA during COVID-19 was lower compared to normal days [45]. A significant reduction of PA during COVID is also reported in a Brazilian study [5], Spanish online survey [68] and Chinese study [69] using the International Physical Activity Questionnaire Short Form (IPAQ-SF) and the Profile of Mood States (POMS).

To our knowledge, this is the first study using the standardized PAQ-C to compare PA pre-COVID and during-COVID lockdown. The PAQ-C/cz questionnaire was recently validated [38] and the pre-COVID raw data were compared with during-COVID raw data collected during the same time of year (November-January). However, a limitation of PAQ-C is that the questionnaire does not offer detailed information about the intensity and time engaged in PA. Therefore, we combined the PAQ-C data with the number of steps reported by 35 subjects who had pedometers available. The use of pedometers is historically the oldest but still currently the most widespread way of instrumental PA monitoring [6, 70]. It is the suggested method to monitor PA to follow prescribed public health guidelines [71]. Although boys reported higher numbers of daily steps, due to the small sample size comparing only 12 boys with 23 girls we have identified no statistical difference between boys and girls. However, this trend is similar to normative data. Simply comparing the current dataset of Czech children’s steps/day with previously published normative data, large differences are noted, which are concerning. It is typically noted that the number of steps/day peak before the age of 12 and slowly decrease throughout adolescence to approximately 8.000–9.000 steps/day by 18 years old. In children, boys typically average 12.000–16.000 steps/day, whereas girls average 10.000-13.000 steps/day [56]. The limited cohort in this study reported boys averaged 7.768 steps/day, and girls averaged only 6.982 steps/day. This concerning trend requires further investigation.

There are some limitations to this study. Only 98 children completed the questionnaire with only 35 also reporting the number of daily steps using smart watch/phones. Employment of such devices may have represented a certain motivation for children to take a larger number of steps. We expect that these children walked more than the rest of the cohort. Therefore, the average number of the steps in the whole cohort was most likely smaller then reported above. The data collection started at the time when some outdoor organized sport activities were still allowed (early November) while children who completed the survey later in December were protected from all organized sport activities. So, during the time of data collection the restriction orders kept slightly changing. This could possibly affect results. Another limitation could be the fact that the study may not fully represent the population as a whole. Parents who are not upset by the lockdown are perhaps not as motivated to complete a questionnaire regarding their child’s lack of PA.

The authors of the study encourage researchers from other countries to use the internationally standardized PAQ-C to conduct surveys in their countries and compare the results internationally, to help establish optimal strategies for preventing detrimental effects of long lasting hypomobility in school-aged children.

## Conclusions

The "second wave" of the COVID-19 pandemic restrictions had a negative impact on PA of Czech boys and girls 8–12 years old. Based on comparison of Czech and international PAQ-C data, it seems that even under normal conditions Czech children are less physically active than their peers abroad. Further significant reduction of children’s PA due to epidemic restriction is alarming. This topic should be considered a public health concern. School, sport and government authorities need to set up effective strategies promoting school children’s PA both during and after COVID.

## Supporting information

S1 Table Study characteristics comparing Cuberek et al. [38] Pre-COVID data with during COVID data of Czech children. (PDF)

S2 Table Comparison of Czech children scores on the PAQ-C regarding PA before and during COVID pandemic (mean [standard deviation]). (PDF)

S3 Table Gender specific scores on the PAQ-C before and during COVID pandemic (mean [standard deviation]). (PDF)

S1 File Master data: Pre-COVID and COVID. (XLSX)

S2 File The Physical Activity Questionnaire for Older Children—the Czech version. (PDF)

S3 File (DOCX)

S1 Graph Comparation of PAQ-C/CZ questionnaire results between Pre-COVID (n = 206) and COVID data (n = 98). PAQ-C–total PAQ-C score; Q1—Spare time activity; Q2—Before school activity, Q3—Physical education; Q4—Recesses; Q5—After school activity; Q6 –Evening activity; Q7 –Weekend activity; Q8 –Statement; Q9 –Weekly activity.
(TIF)

S2 Graph Spare time activity during COVID lockdown–reported mean values for each type of PA (n = 98), frequency of individual sort of PA from question 1 (evaluation of these leisure activities is a standard part of PAQ-C). (TIF)

## References

[1] Jakubec L, Dygrýn J, Šimůnek A, Frömel K. Validita originálního algoritmu pro odhad pohybové aktivity a sedavého chování z dotazníku Youth Activity Profile u českých dětí a adolescentů. Tělesná kultura. 2020;42: 62–69.

[2] Kaptoge S, Pennells L, De Bacquer D, Cooney MT, Kavousi M, Stevens G, et al. World Health Organization cardiovascular disease risk charts: revised models to estimate risk in 21 global regions. The Lancet Global Health. 2019;7: e1332–e1345. doi: 10.1016/S2214-109X(19)30318-3 31488387 PMC7025029

[3] Nemet D. Childhood Obesity, Physical Activity, and Exercise. Pediatric exercise science. 2016;28: 48–51. doi: 10.1123/pes.2016-0014 26887608

[4] Tremblay MS, Carson V, Chaput J-P, Connor Gorber S, Dinh T, Duggan M, et al. Canadian 24-hour movement guidelines for children and youth: an integration of physical activity, sedentary behaviour, and sleep. Applied Physiology, Nutrition, and Metabolism. 2016;41: S311–S327. 10.1139/apnm-2016-0151 27306437

[5] Sá C dos SC de, Pombo A, Luz C, Rodrigues LP, Cordovil R. COVID-19 social isolation in brazil: effects on the physical activity routine of families with children. Rev paul pediatr. 2021;39: e2020159. doi: 10.1590/1984-0462/2021/39/2020159 33206868 PMC7659029

[6] Sigmund E., Sigmundová D., & Šnoblová R. Monitorování lokomoční pohybové aktivity dětí pomocí pedometrů: přesnost, doporučení a praktické příklady. Medicina Sportiva Bohemica et Slovaca. 2011;20: 17–23.

[7] Miles L. Physical activity and health. Nutrition bulletin. 2007;32: 314–363. doi: 10.1016/j.healthpol.2007.03.004 17498835

[8] Anderson PM, Butcher KF. Childhood obesity: trends and potential causes. The Future of children. 2006; 19–45. doi: 10.1353/foc.2006.0001 16532657

[9] Fox KR. The influence of physical activity on mental well-being. Public health nutrition. 1999;2: 411–418. doi: 10.1017/s1368980099000567 10610081

[10] Guo Y-R, Cao Q-D, Hong Z-S, Tan Y-Y, Chen S-D, Jin H-J, et al. The origin, transmission and clinical therapies on coronavirus disease 2019 (COVID-19) outbreak–an update on the status. Military Med Res. 2020;7: 11. doi: 10.1186/s40779-020-00240-0 32169119 PMC7068984

[11] López-Bueno R, López-Sánchez GF, Casajús JA, Calatayud J, Tully MA, Smith L. Potential health-related behaviors for pre-school and school-aged children during COVID-19 lockdown: A narrative review. Preventive Medicine. 2021;143: 106349. doi: 10.1016/j.ypmed.2020.106349 33271236 PMC7701882

[12] Maltoni G, Zioutas M, Deiana G, Biserni GB, Pession A, Zucchini S. Adolescent males suffered from reduced physical activity and increased BMI during COVID- 19 pandemic. Nutrition, Metabolism and Cardiovascular Diseases. 2021; S093947532100140X. doi: 10.1016/j.numecd.2021.03.018 33994065

[13] Beck AL, Huang JC, Lendzion L, Fernandez A, Martinez S. Impact of the COVID-19 pandemic on parents’ perception of health behaviors in children with overweight and obesity. Academic Pediatrics. 2021; S1876285921002679. doi: 10.1016/j.acap.2021.05.015 34023488 PMC8561012

[14] Law EF, Zhou C, Seung F, Perry F, Palermo TM. Longitudinal study of early adaptation to the coronavirus disease pandemic among youth with chronic pain and their parents: effects of direct exposures and economic stress. Pain. 2021;Publish Ahead of Print. doi: 10.1097/j.pain.0000000000002290 PMC8205975 34050112

[15] Zolnikov TR, Clark T, Zolnikov T. Likely Exacerbation of Psychological Disorders from Covid-19 Response. J Prim Care Community Health. 2021;12: 215013272110167. doi: 10.1177/21501327211016739 33998328 PMC8132076

[16] Li SH, Beames JR, Newby JM, Maston K, Christensen H, Werner-Seidler A. The impact of COVID-19 on the lives and mental health of Australian adolescents. Eur Child Adolesc Psychiatry. 2021 [cited 4 Jun 2021]. doi: 10.1007/s00787-021-01790-x 33913036 PMC8080862

[17] Panda PK, Gupta J, Chowdhury SR, Kumar R, Meena AK, Madaan P, et al. Psychological and Behavioral Impact of Lockdown and Quarantine Measures for COVID-19 Pandemic on Children, Adolescents and Caregivers: A Systematic Review and Meta-Analysis. Journal of Tropical Pediatrics. 2021;67: fmaa122. doi: 10.1093/tropej/fmaa122 33367907 PMC7798512

[18] Bucak IH, Almis H, Tasar SO, Uygun H, Turgut M. Have the sleep habits in children of health workers been more affected during the COVID-19 pandemic? Sleep Medicine. 2021;83: 235–240. doi: 10.1016/j.sleep.2021.05.003 34049042 PMC9759934

[19] Perez D, Thalken JK, Ughelu NE, Knight CJ, Massey WV. Nowhere to Go: Parents’ Descriptions of Children’s Physical Activity During a Global Pandemic. Front Public Health. 2021;9: 642932. doi: 10.3389/fpubh.2021.642932 33981665 PMC8107355

[20] López-Bueno R, Calatayud J, Andersen LL, Casaña J, Ezzatvar Y, Casajús JA, et al. Cardiorespiratory fitness in adolescents before and after the COVID-19 confinement: a prospective cohort study. Eur J Pediatr. 2021 [cited 4 Jun 2021]. doi: 10.1007/s00431-021-04029-8 33733288 PMC7968551

[21] Gaba A, Rubin L, Sigmund E, Badura P, Dygryn J, Kudlacek M, et al. Executive summary of the Czech Republic’s 2018 Report Card on Physical Activity for Children and Youth. Acta Gymnica. 2019;49: 92–102. doi: 10.5507/ag.2019.007

[22] World Health Organization. CZECH REPUBLIC PHYSICAL ACTIVITY FACTSHEET. Available: https://ec.europa.eu/assets/eac/sport/library/factsheets/czech-rep-factsheet_en.pdf

[23] Komenda M, Bulhart V, Karolyi M, Jarkovský J, Mužík J, Májek O, et al. Complex Reporting of the COVID-19 Epidemic in the Czech Republic: Use of an Interactive Web-Based App in Practice. J Med Internet Res. 2020;22: e19367. doi: 10.2196/19367 32412422 PMC7254961

[24] Johns Hopkins University & Medicine. MORTALITY ANALYSES. In: Johns Hopkins University & Medicine [Internet]. Available: https://origin-coronavirus.jhu.edu/data/mortality

[25] Hesketh KR, Lakshman R, van Sluijs EM. Barriers and facilitators to young children’s physical activity and sedentary behaviour: a systematic review and synthesis of qualitative literature. Obesity Reviews. 2017;18: 987–1017. doi: 10.1111/obr.12562 28589678 PMC5575514

[26] Schmidt SCE, Anedda B, Burchartz A, Eichsteller A, Kolb S, Nigg C, et al. Physical activity and screen time of children and adolescents before and during the COVID-19 lockdown in Germany: a natural experiment. Sci Rep. 2020;10: 21780. doi: 10.1038/s41598-020-78438-4 33311526 PMC7733438

[27] UNESCO 2021. Education: From disruption to recovery. In: UNESCO.

[28] Fillon A, Genin P, Larras B, Vanhelst J, Luiggi M, Aubert S, et al. France’s 2020 Report Card on Physical Activity and Sedentary Behaviors in Children and Youth: Results and Progression. Journal of Physical Activity and Health. 2021; 1–7. doi: 10.1123/jpah.2021-0025 34039777

[29] Pombo A, Luz C, Rodrigues LP, Ferreira C, Cordovil R. Correlates of children’s physical activity during the COVID-19 confinement in Portugal. Public Health. 2020;189: 14–19. doi: 10.1016/j.puhe.2020.09.009 33126117 PMC7508519

[30] Cachón-Zagalaz J, Zagalaz-Sánchez Ma L, Arufe-Giráldez V, Sanmiguel-Rodríguez A, González-Valero G. Physical Activity and Daily Routine among Children Aged 0–12 during the COVID-19 Pandemic in Spain. IJERPH. 2021;18: 703. doi: 10.3390/ijerph18020703 33467514 PMC7830357

[31] Schnaiderman D, Bailac M, Borak L, Comar H, Eisner A, Ferrari A, et al. Psychological impact of COVID-19 lockdown in children and adolescents from San Carlos de Bariloche, Argentina: Parents’ perspective. Arch Argent Pediat. 2021;119. doi: 10.5546/aap.2021.eng.170 34033416

[32] Kowalski KC, Crocker PR, Kowalski NP. Convergent validity of the physical activity questionnaire for adolescents. Pediatric exercise science. 1997;9: 342–352.

[33] Sallis JF, Saelens BE. Assessment of physical activity by self-report: status, limitations, and future directions. Research quarterly for exercise and sport. 2000;71: 1–14. 10.1080/02701367.2000.11082780 25680007

[34] Welk GJ, Wood K. Physical activity assessments in physical education: A practical review of instruments and their use in the curriculum. Journal of Physical Education, Recreation & Dance. 2000;71: 30–40. 18858293

[35] Colley RC, Butler G, Garriguet D, Prince SA, Roberts KC. Comparison of self-reported and accelerometer-measured physical activity among Canadian youth. Health Reports. 2019;30: 3–12. doi: 10.25318/82-003-x201900700001-eng 31314124

[36] Crocker PR, Bailey D, Faulkner R, Kowalski K, McGrath R. Measuring general levels of physical activity: preliminary evidence for the Physical Activity Questionnaire for Older Children. Medicine & Science in Sports & Exercise. 1997;29: 1344–1349. doi: 10.1097/00005768-199710000-00011 9346166

[37] Rubín L, Mitáš J, Dygrýn J, Vorlíček M, Nykodým J, Řepka E, et al. Pohybová aktivita a tělesná zdatnost českých adolescentů v kontextu zastavěného prostředí. Univerzita Palackého v Olomouci; 2018.

[38] Cuberek R, Janíková M, Dygrýn J. Adaptation and validation of the Physical Activity Questionnaire for Older Children (PAQ-C) among Czech children. PloS one. 2021;16: e0245256. doi: 10.1371/journal.pone.0245256 33434223 PMC7802927

[39] Marques ES, Moraes CL de, Hasselmann MH, Deslandes SF, Reichenheim ME. Violence against women, children, and adolescents during the COVID-19 pandemic: overview, contributing factors, and mitigating measures. Cadernos de saude publica. 2020;36: e00074420. doi: 10.1590/0102-311X00074420 32374808

[40] Kowalski KC, Crocker PR, Donen RM. The physical activity questionnaire for older children (PAQ-C) and adolescents (PAQ-A) manual. College of Kinesiology, University of Saskatchewan. 2004;87: 1–38.

[41] Cohen J. Statistical power analysis for the behavioural sciences. Hillsdale, NJ: Laurence Erlbaum Associates. Inc; 1988.

[42] Geroge D, Mallery P. SPSS for windows step by step: A simple guide and reference. Boston: Allyn & Bacon; 2003.

[43] Xiang S, Dong J, Li X, Li L. Association between Sleep Duration, Physical Activity, and Mental Health Disorders: A Secondary Analysis of the National Survey of Children’s Health 2017–2018. Boffano P, editor. BioMed Research International. 2021;2021: 1–7. doi: 10.1155/2021/5585678 33816615 PMC7987431

[44] Filos D, Lekka I, Kilintzis V, Stefanopoulos L, Karavidopoulou Y, Maramis C, et al. Exploring associations between children’s obesogenic behaviours and local environment using big data (Preprint). JMIR mHealth and uHealth. 2020 [cited 7 Jun 2021]. doi: 10.2196/26290 34048353 PMC8274675

[45] Pombo A, Luz C, Rodrigues LP, Ferreira C, Cordovil R. Correlates of Children’s Physical Activity During the Covid-19 Confinement in Portugal. In Review; 2020 Jul. doi: 10.1016/j.puhe.2020.09.009 33126117 PMC7508519

[46] Vuković J, Matić RM, Milovanović IM, Maksimović N, Krivokapić D, Pišot S. Children’s Daily Routine Response to COVID-19 Emergency Measures in Serbia. Front Pediatr. 2021;9: 656813. doi: 10.3389/fped.2021.656813 33959575 PMC8093502

[47] McGall SE, McGuigan MR, Nottle C. Contribution of free play towards physical activity guidelines for New Zealand primary school children aged 7–9 years. British journal of sports medicine. 2011;45: 120–124. doi: 10.1136/bjsm.2009.065318 19696036

[48] Ng K, Cosma A, Svacina K, Boniel-Nissim M, Badura P. Czech adolescents’ remote school and health experiences during the spring 2020 COVID-19 lockdown. Preventive Medicine Reports. 2021;22: 101386. doi: 10.1016/j.pmedr.2021.101386 34012765 PMC8113712

[49] Vilchez JA, Kruse J, Puffer M, Dudovitz RN. Teachers and School Health Leaders’ Perspectives on Distance Learning Physical Education During the COVID ‐19 Pandemic. J School Health. 2021; josh.13030. doi: 10.1111/josh.13030 34031879 PMC8207123

[50] Gobbi E, Maltagliati S, Sarrazin P, di Fronso S, Colangelo A, Cheval B, et al. Promoting Physical Activity during School Closures Imposed by the First Wave of the COVID-19 Pandemic: Physical Education Teachers’ Behaviors in France, Italy and Turkey. IJERPH. 2020;17: 9431. doi: 10.3390/ijerph17249431 33339228 PMC7767079

[51] ErdiM L, Ergün A, Kuğuoğlu S. Reliability and validity of the Turkish version of the Physical Activity Questionnaire for Older Children (PAQ-C). Turk J Med Sci. 2019;49: 162–169. doi: 10.3906/sag-1806-212 30764593 PMC7371234

[52] Thomas EL, Upton D. Psychometric properties of the physical activity questionnaire for older children (PAQ-C) in the UK. Psychology of Sport and Exercise. 2014;15: 280–287. doi: 10.1016/j.psychsport.2014.02.002

[53] Jing WJ, Tom B, Patrick LW, An CT, Jane PA. Validation of the Physical Activity Questionnaire for Older Children (PAQ-C) among Chinese Children. Biomed Environ Sci. 2016;29: 177–86. doi: 10.3967/bes2016.022 27109128

[54] Trost SG, Sallis JF, Pate RR, Freedson PS, Taylor WC, Dowda M. Evaluating a model of parental influence on youth physical activity. American Journal of Preventive Medicine. 2003;25: 277–282. doi: 10.1016/s0749-3797(03)00217-4 14580627

[55] Rowlands AV, Eston RG, Ingledew DK. Relationship between activity levels, aerobic fitness, and body fat in 8-to 10-yr-old children. Journal of Applied Physiology. 1999;86: 1428–1435. doi: 10.1152/jappl.1999.86.4.1428 10194232

[56] Tudor-Locke C, Craig CL, Beets MW, Belton S, Cardon GM, Duncan S, et al. How many steps/day are enough? for children and adolescents. International Journal of Behavioral Nutrition and Physical Activity. 2011;8: 1–14. doi: 10.1186/1479-5868-8-78 21798014 PMC3166269

[57] Vincent S. D., & Pangrazi R. P. An examination of the activity patterns of elementary school children. ediatric Exercise Science. 2002;14: 432–441. 10.1123/pes.14.4.432

[58] Panel NE. Third report of the NCEP. Expert panel of detection, evaluation and treatment of high blood cholesterol in adults (ATP III): NIH Publication. Bethesda: National Heart. Lung and Blood Institute. 2001.

[59] Moore JB, Jr JCH, Barbeau P, Gutin B, Treviño RP. Validation of the Physical Activity Questionnaire for Older Children in Children of Different Races. Pediatric Exercise Science. 2007;19: 6–19. doi: 10.1123/pes.19.1.6 17554153

[60] Twisk JW. Physical activity guidelines for children and adolescents. Sports medicine. 2001;31: 617–627. doi: 10.2165/00007256-200131080-00006 11475323

[61] Janssen I. Physical activity guidelines for children and youth. Applied Physiology, Nutrition, and Metabolism. 2007;32: S109–121. 18213942

[62] Tremblay MS, Warburton DE, Janssen I, Paterson DH, Latimer AE, Rhodes RE, et al. New Canadian physical activity guidelines. Applied physiology, nutrition, and metabolism. 2011;36: 36–46. doi: 10.1139/H11-009 21326376

[63] Singh K, Tripathi K. Physical Activity Guidelines for Children and Youth. Available at SSRN 2308560. 2013.

[64] Corbin CB, Pangrazi RP. Guidelines for appropriate physical activity for elementary school children. Update Reston, VA: NASPE Publications. 2003.

[65] Máček M, Máčková J, Smolíková L. Počet kroků jako ukazatel tělesné zdatnosti. Medicina Sportiva Bohemica et Slovaca. 2010;19.

[66] Dunton GF, Do B, Wang SD. Early effects of the COVID-19 pandemic on physical activity and sedentary behavior in children living in the U.S. BMC Public Health. 2020;20: 1351. doi: 10.1186/s12889-020-09429-3 32887592 PMC7472405

[67] Moore SA, Faulkner G, Rhodes RE, Brussoni M, Chulak-Bozzer T, Ferguson LJ, et al. Impact of the COVID-19 virus outbreak on movement and play behaviours of Canadian children and youth: a national survey. Int J Behav Nutr Phys Act. 2020;17: 85. doi: 10.1186/s12966-020-00987-8 32631350 PMC7336091

[68] López-Bueno R, López-Sánchez GF, Casajús JA, Calatayud J, Gil-Salmerón A, Grabovac I, et al. Health-related behaviors among school-aged children and adolescents during the Spanish Covid-19 confinement. Frontiers in pediatrics. 2020;8. doi: 10.3389/fped.2020.00008 33042917 PMC7516648

[69] Zhang X, Zhu W, Kang S, Qiu L, Lu Z, Sun Y. Association between physical activity and mood states of children and adolescents in social isolation during the CoViD-19 epidemic. International journal of environmental research and public health. 2020;17: 7666. doi: 10.3390/ijerph17207666 33096659 PMC7589310

[70] Rowe DA, Mahar MT, Raedeke TD, Lore J. Measuring physical activity in children with pedometers: Reliability, reactivity, and replacement of missing data. Pediatric Exercise Science. 2004;16: 343–354.

[71] Adams MA, Johnson WD, Tudor-Locke C. Steps/day translation of the moderate-to-vigorous physical activity guideline for children and adolescents. International Journal of Behavioral Nutrition and Physical Activity. 2013;10: 1–11. doi: 10.1186/1479-5868-10-49 23601369 PMC3639120

