# Supplementary material for: The impact of COVID-19 on Physical Activity of Czech children
Source: PLoS One. 2021 Jul 8;16(7):e0254244. doi: 10.1371/journal.pone.0254244 (PMC8266068; doi:10.1371/journal.pone.0254244)
Supplement: S1 Table — (PDF) [file pone.0254244.s001.pdf]

Table 1. Study characteristics comparing Cuberek et al. (38) Pre-COVID data with during COVID data of Czech children.

	**Sex**	**Sample Size**	**Age***	**BMI***
Cuberek et al Pre-COVID cohort	Male	106	11.08 (0.84)	18.46 (3.13)
	Female	100	11.17 (0.82)	17.36 (2.68)
	Total	206	11.13 (0.83)	17.92 (2.97)
During COVID cohort	Male	42	10.21 (1.49)	17.56 (3.06)
	Female	56	10.02 (1.46)	17.20 (2.70)
	Total	98	10.10 (1.47)	17.35 (2.85)

*Reported as mean (standard deviation), note: BMI: Body mass index
