# Supplementary material for: The impact of COVID-19 on Physical Activity of Czech children
Source: PLoS One. 2021 Jul 8;16(7):e0254244. doi: 10.1371/journal.pone.0254244 (PMC8266068; doi:10.1371/journal.pone.0254244)
Supplement: S2 Table — (PDF) [file pone.0254244.s002.pdf]

Table 2. Comparison of Czech children scores on the PAQ-C regarding PA before and during COVID pandemic (Mean [Standard Deviation]).

Measure	Cuberek et al. Pre-COVID (n = 206)	COVID Lockdown (n= 98)	Mean Difference (95% CI)	Effect Size	*P* Value
Total PAQ-C Score	2.69 (0.59)	2.30 (0.66)	0.38 (0.24, 0.53)	0.63	<.001*
Q1 Spare time activity	1.34 (0.22)	1.26 (0.17)	0.07 (0.03, 0.12)	0.38	.001**
Q2 Before-school activity	2.06 (1.37)	1.63 (1.08)	0.43 (0.15, 0.72)	0.34	.003**
Q3 Physical education	3.83 (1.15)	2.26 (1.37)	1.57 (1.26, 1.89)	1.28	<.001**
Q4 Recesses	2.82 (0.95)	1.87 (1.03)	0.95 (0.71, 1.18)	0.97	<.001**
Q5 After-school activity	3.00 (1.11)	3.14 (1.19)	-0.14 (-0.42, 0.14)	-0.12	0.32
Q6 Evenings	2.59 (1.07)	2.43 (1.30)	0.16 (-0.11, 0.44)	0.14	0.25
Q7 Weekend	2.90 (0.98)	2.82 (0.92)	0.08 (-0.15, 0.31)	0.09	0.49
Q8 Statement	2.71 (1.04)	2.65 (1.10)	0.06 (-0.19, 0.32)	0.06	0.64
Q9 Weekly activity	2.93 (0.76)	2.70 (0.85)	0.23 (0.04, 0.43)	0.30	0.016

*Statistically significant difference observed ($P < 0.05$)

**Statistically significant difference observed (Bonferroni correction $P < 0.005$)

Note: PAQ-C: Physical Activity Questionnaire for Older Children

Values are tabulated scores from PAQ-C

Effect size = calculated Cohen's d
