# Supplementary material for: The impact of COVID-19 on Physical Activity of Czech children
Source: PLoS One. 2021 Jul 8;16(7):e0254244. doi: 10.1371/journal.pone.0254244 (PMC8266068; doi:10.1371/journal.pone.0254244)
Supplement: S3 Table — (PDF) [file pone.0254244.s003.pdf]

Table 3. Gender specific scores on the PAQ-C before and during COVID pandemic (Mean [Standard Deviation]).

**Measure**		**Cuberek et al. Pre-COVID**	**COVID Lockdown**	**Mean Difference (95% CI)**	**Effect Size**	***P* V*alue***
Total PAQ Score	Male	2.69 (0.62)	2.32 (0.69)	0.37 (0.14, 0.61)	0.59	.002*
	Female	2.68 (0.56)	2.29 (0.64)	0.39 (0.20, 0.58)	0.66	<.001*

*Statistically significant difference observed (Bonferroni correction $P < 0.025$)

Note: PAQ-C: Physical Activity Questionnaire for Older Children
Values are tabulated scores from PAQ-C
Effect size = calculated Cohen's d
