# Supplementary material for: The impact of COVID-19 on Physical Activity of Czech children
Source: PLoS One. 2021 Jul 8;16(7):e0254244. doi: 10.1371/journal.pone.0254244 (PMC8266068; doi:10.1371/journal.pone.0254244)
Supplement: S2 File — (PDF) [file pone.0254244.s005.pdf]

Dotazník pohybové aktivity dětí
(PAQ-C/cz)

Milá žákyně, milý žáku,
prosíme o vyplnění dotazníku. Dotazník je anonymní. Pokus se zodpovědět všechny otázky upřímně a jak nejlépe dovedeš – je to pro nás velmi důležité. Cílem dotazníku je zjistit úroveň tvé pohybové aktivity v posledních 7 dnech.

PAMATUJ:

1. KŘÍŽKUJ – Své odpovědi označuj křížkem ☒
2. CHYBY – Pokud spleteš odpověď, chybnou zaškrtej ☒ a správnou nově označ křížkem.
3. POHYBOVÁ AKTIVITA – Jde o různé sporty, tanec, všechny pohybové hry, běžná jízda na kole, chození do školy, se psem, po obchodě, na houbách…, běhání, skákání, lezení, různé práce na zahradě a podobně.
4. 7 DNÍ – V dotazníku se ptáme na pohybové aktivity za posledních 7 dní. Zkus si je vybavit.
5. ŽÁDNÝ TEST – V dotazníku nejsou správné či špatné odpovědi. Nejedná se o žádný test. Nebude to známkováno.

OSOBNÍ ÚDAJE

Pohlaví	☐ dívka	☐ chlapec
Věk	_________ let	
Tělesná výška	_________ cm	
Tělesná hmotnost	_________ kg	

---

1. V posledních 7 dnech: Kterým aktivitám ses věnoval nepřetržitě alespoň půl hodiny? Kolikrát během 7 dní to bylo?
   KROK 1: v celém sloupci označ křížkem, kterým aktivitám ses věnoval (mimo tělocvik).
   KROK 2: označ křížkem, jak často ses svým aktivitám věnoval v posledních 7 dnech.

**KROK 1** ANO	**KROK 2** 1–2krát	3–4krát	5–6krát	7krát a více
☐ Atletika (běhání, skákání, házení)	☐	☐	☐	☐
☐ Basketbal	☐	☐	☐	☐
☐ Bojové sporty (judo, karate a jiné)	☐	☐	☐	☐
☐ Florbal	☐	☐	☐	☐
☐ Fotbal	☐	☐	☐	☐
☐ Gymnastika	☐	☐	☐	☐
☐ Házená, vybíjená	☐	☐	☐	☐
☐ In-line bruslení, bruslení na ledě	☐	☐	☐	☐
☐ Jízda na kole, koloběžce (ne e-kolo a e-koloběžka)	☐	☐	☐	☐
☐ Jízda na koni	☐	☐	☐	☐
☐ Lední hokej	☐	☐	☐	☐
☐ Parkour, street workout	☐	☐	☐	☐
☐ Plavání a jiné aktivity ve vodě	☐	☐	☐	☐
☐ Posilovací cvičení, fitness, jóga	☐	☐	☐	☐
☐ Lyžování, běžky, snowboarding	☐	☐	☐	☐
☐ Skateboarding, penny board	☐	☐	☐	☐
☐ Softball, baseball	☐	☐	☐	☐
☐ Tanec, aerobik, hip-hop, street-dance	☐	☐	☐	☐
☐ Tenis, squash, stolní tenis, soft-tenis, badminton	☐	☐	☐	☐
☐ Turistika, delší chůze	☐	☐	☐	☐
☐ Volejbal, beach volejbal	☐	☐	☐	☐
☐ Jiné	☐	☐	☐	☐

2. V posledních 5 školních dnech: V kolika dnech ses *ráno před školou* věnoval nějakému sportu, hraní her nebo jiným pohybovým aktivitám, u kterých jsi byl velmi aktivní (hodně ses u nich zadýchal, zpotil a unavil)?
(Označ křížkem pouze jednu odpověď.)

- ☐ v žádném dni;
- ☐ v 1 dni;
- ☐ ve 2 nebo 3 dnech;
- ☐ ve 4 dnech;
- ☐ v 5 dnech.

3. V posledních 5 školních dnech: Kolikrát jsi byl *v tělocviku* velmi aktivní? Velmi aktivní je intenzivní hraní, běhání, skákání, házení, plavání, u kterého jsi byl hodně zadýchaný a zpocený.
(Označ křížkem pouze jednu odpověď.)

- ☐ neměl jsem tělocvik nebo jsem necvičil;
- ☐ málokdy;
- ☐ občas;
- ☐ docela často;
- ☐ skoro pořád nebo pořád.

4. V posledních 5 školních dnech: Co jsi dělal po většinu času *o všech přestávkách* ve škole? Počítej zde i dobu mezi příchodem do školy a začátkem vyučování.
(Označ křížkem pouze jednu odpověď.)

- ☐ seděl (povídal, četl, plnil školní povinnosti);
- ☐ postával jsem nebo se pomalu procházel;
- ☐ trochu jsem pobíhal nebo si hrál (bez výraznějšího zadýchání);
- ☐ docela hodně jsem pobíhal nebo si hrál (zadýchal jsem se víc, než při běžné chůzi);
- ☐ po většinu času jsem intenzivně běhal nebo si hrál (hodně jsem se zadýchal a zpotil).

5. V posledních 5 školních dnech: V kolika dnech ses *hned po škole a odpoledne* věnoval nějakému sportu, hraní her nebo jiným pohybovým aktivitám, u kterých jsi byl velmi aktivní (hodně ses zadýchal nebo zpotil)?
Jedná se o dobu mezi odchodem z budovy školy a přibližně 6 hodinou večer.
(Označ křížkem pouze jednu odpověď.)

- ☐ v žádném dni;
- ☐ v 1 dni;
- ☐ ve 2 nebo 3 dnech;
- ☐ ve 4 dnech;
- ☐ v 5 dnech.

6. V posledních 7 dnech: V kolika dnech ses *navečer* věnoval nějakému sportu, hraní her nebo jiným pohybovým aktivitám, u kterých jsi byl velmi aktivní (hodně ses zadýchal nebo zpotil)?
Navečer se rozumí doba mezi 6 hodinou večer a spánkem.
(Označ křížkem pouze jednu odpověď.)

- ☐ v žádném dni;
- ☐ v 1 dni;
- ☐ ve 2 nebo 3 dnech;
- ☐ ve 4 nebo 5 dnech;
- ☐ v 6 nebo 7 dnech.

7. Během víkendu: Kolikrát ses věnoval nějakému sportu, hraní her nebo jiným pohybovým aktivitám, u kterých jsi byl velmi aktivní (hodně ses zadýchal nebo zpotil)?
(Označ křížkem pouze jednu odpověď.)

- ☐ vůbec;
- ☐ 1krát;
- ☐ 2 – 3krát;
- ☐ 4 – 5krát;
- ☐ 6 a vícekrát.

8. V posledních 7 dnech: Která z následujících vět nejlépe popisuje, co jsi během posledních 7 dní dělal?
Nejdříve si přečti všechny odpovědi. Potom vyber a označ křížkem pouze tu, která Tě nejvíc vystihuje.

- ☐ Všechen nebo většinu svého volného času jsem se věnoval aktivitám, které vyžadovaly malé fyzické úsilí.
- ☐ Občas (1–2krát za poslední týden) jsem se ve svém volném čase věnoval pohybovým aktivitám, u kterých jsem byl hodně zadýchaný a zpocený.
- ☐ Často (3–4krát) jsem se ve svém volném čase věnoval pohybovým aktivitám, u kterých jsem byl hodně zadýchaný a zpocený.
- ☐ Docela často (5–6krát) jsem se ve svém volném čase věnoval pohybovým aktivitám, u kterých jsem byl hodně zadýchaný a zpocený.
- ☐ Velmi často (7 nebo vícekrát) jsem se ve svém volném čase věnoval pohybovým aktivitám, u kterých jsem byl hodně zadýchaný a zpocený.

9. V posledních 7 dnech: Označ, jak často ses během celého dne věnoval pohybovým aktivitám. Pozor na pořadí dnů v tabulce! Příklad: pokud je dnes čtvrtek, pak se ptáme na minulý čtvrtek až včerejší středu.
(V každém řádku označ křížkem pouze jednu odpověď.)

	nikdy	občas	středně často	často	velmi často
Pondělí	☐	☐	☐	☐	☐
Úterý	☐	☐	☐	☐	☐
Středa	☐	☐	☐	☐	☐
Čtvrtek	☐	☐	☐	☐	☐
Pátek	☐	☐	☐	☐	☐
Sobota	☐	☐	☐	☐	☐
Neděle	☐	☐	☐	☐	☐

10. V posledních 7 dnech: Byl jsi v průběhu posledních 7 dní nemocný nebo ti něco jiného bránilo věnovat se pohybovým aktivitám, kterým se normálně věnuješ?
(Označ křížkem pouze jednu odpověď.)

- ☐ ANO
  Napiš, co ti bylo: ______________________________
  ____
- ☐ NE

Nyní se ještě jednou podívej, zda jsi skutečně odpověděl na všechny otázky.

DĚKUJEME ZA VYPLNĚNÍ DOTAZNÍKU.
