# Supplementary material for: The impact of COVID-19 on Physical Activity of Czech children
Source: PLoS One. 2021 Jul 8;16(7):e0254244. doi: 10.1371/journal.pone.0254244 (PMC8266068; doi:10.1371/journal.pone.0254244)
Supplement: S3 File — (DOCX) [file pone.0254244.s006.docx]

**S3 File:** available online at: <https://journals.plos.org/plosone/article?id=10.1371/journal.pone.0245256>
