# Supplementary figures and images for: The impact of COVID-19 on Physical Activity of Czech children
Source: PLoS One. 2021 Jul 8;16(7):e0254244. doi: 10.1371/journal.pone.0254244 (PMC8266068; doi:10.1371/journal.pone.0254244)

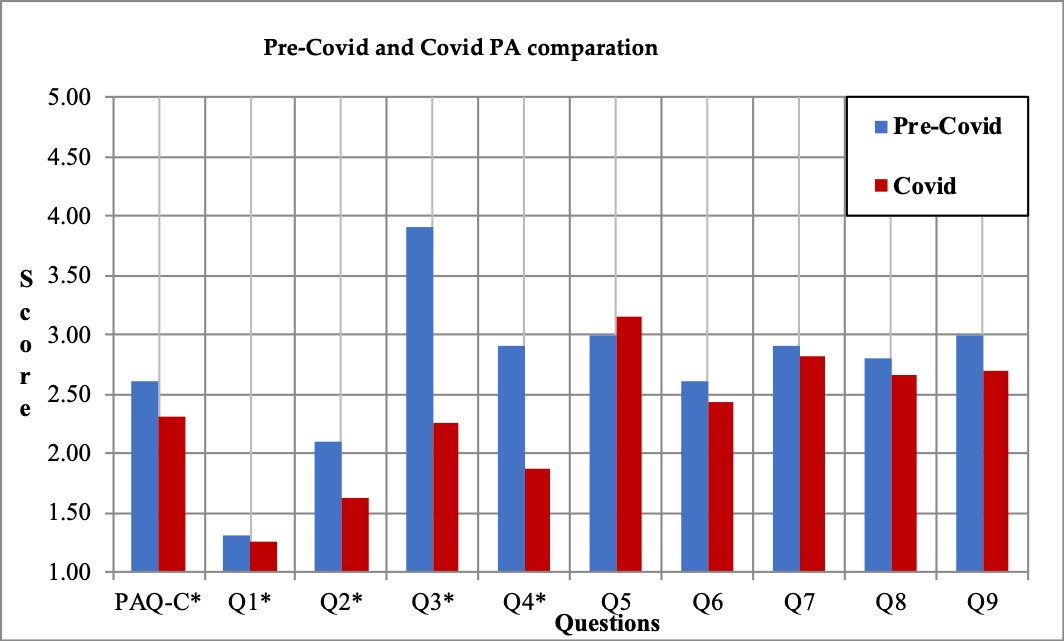

Supplement: S1 Graph — PAQ-C–total PAQ-C score; Q1—Spare time activity; Q2—Before school activity, Q3—Physical education; Q4—Recesses; Q5—After school activity; Q6 –Evening activity; Q7 –Weekend activity; Q8 –Statement; Q9 –Weekly activity. (TIF) [file pone.0254244.s007.tif]

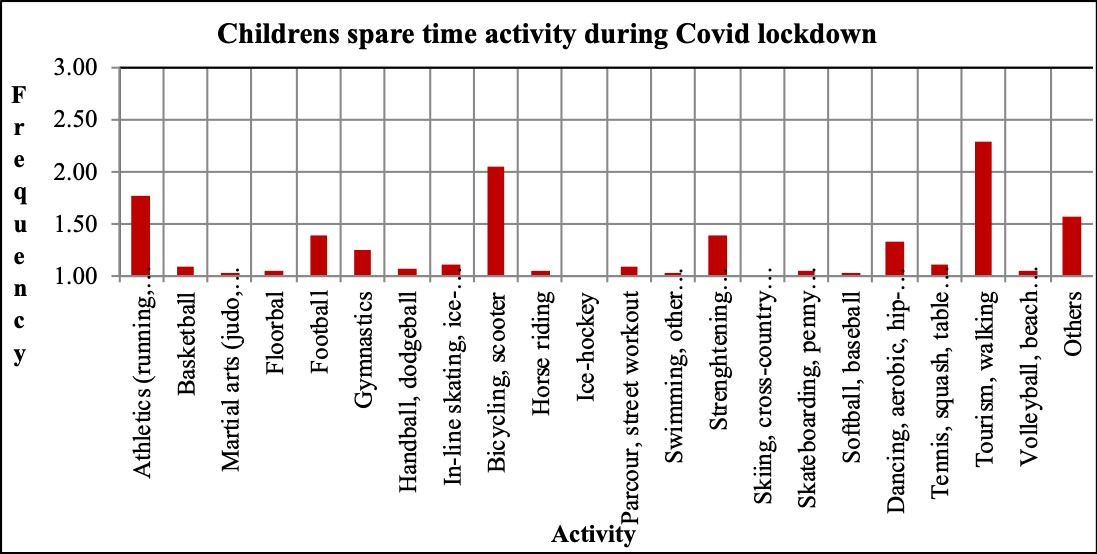

Supplement: S2 Graph — (TIF) [file pone.0254244.s008.tif]
